# Treatment utilization among people with drug use disorders in prison: a national longitudinal cohort study

**DOI:** 10.1186/s40352-024-00302-8

**Published:** 2024-11-26

**Authors:** Nicoline Toresen Lokdam, Marianne Riksheim Stavseth, Ingeborg Skjaervø, Anne Bukten

**Affiliations:** 1 Section for Clinical Addiction Research, Oslo University Hospital, Oslo, Norway; 2 Norwegian Centre for Addiction Research, University of Oslo, Kirkeveien 166, Oslo, 0407 Norway

**Keywords:** Prison, NorMA cohort, Drug use, Drug use disorder, Treatment, DUDIT

## Abstract

**Background:**

Drug use disorders are highly prevalent among people in prison. Nevertheless, treatment coverage for individuals with drug use disorders in prison remains understudied and unknown. The aim of this study was to estimate treatment coverage among a sample of people with harmful and likely drug dependence before entering prison and to investigate the factors associated with treatment utilization.

**Methods:**

This was a longitudinal cohort study using baseline survey data linked to registry data on imprisonment and treatment utilization. The study is based on the Norwegian Offender Mental Health and Addiction (NorMA) cohort (*n* = 733) recruited in 2013–2014 from 57 Norwegian prisons. At baseline, participants reported drug use the year before imprisonment, using the Drug Use Disorder Identification Test (DUDIT). The outcome of interest was DUD treatment, defined as at least one DUD treatment episode from the specialized health services during baseline imprisonment.

**Results:**

40% of the sample had DUDIT scores that indicated likely drug dependence (≥ 25) and a need for treatment. Among this group, 64% received DUD treatment while imprisoned. Longer imprisonment (> 12 months; aOR = 8.87, *p* < 0.001), Nordic country of birth (aOR = 2.85, *p* = 0.003), daily/almost daily injecting drug use (aOR = 2.58, *p* < 0.001) and polydrug use (aOR = 2.19, *p* = 0.002) were positively associated with treatment utilization in prison.

**Conclusions:**

Most people with likely drug dependence before entering prison in Norway received DUD treatment during their time in prison. More severe drug use (Injecting drug use and polydrug use), longer imprisonments and being born in the Nordics were positively associated with treatment utilization. People in prison must have access to adequate treatment during imprisonment, and further studies should investigate the possible individual or structural barriers resulting in treatment gaps, especially for people who are foreign born and people with short sentences.

**Supplementary Information:**

The online version contains supplementary material available at 10.1186/s40352-024-00302-8.

## Introduction

Drug Use Disorders are highly prevalent among people in prison compared to the general population (Carpentier et al., 2018; European Monitoring Centre for Drugs and Drug Addiction, 2022; Fazel et al., 2017; van de Baan et al., 2022; World Health Organization, 2014b). With a growing global prison population (Fair & Walmsley, 2021), addressing the health needs of people in prisons becomes increasingly important to reduce social inequality in health in the total population (Kinner & Young, 2018).

In contrast, the Scandinavian prison population has been decreasing in numbers, while also representing an increasingly selected group, including an increased level of mental health and substance use disorders (Bukten et al., 2024). An increasing burden of disease among people in prison makes research on treatment needs and potential barriers for treatment even more important.

According to the World Health Organization, the goal of drug use disorder treatment in prison must be to improve health and ideally that people are psychosocially stabilized with continued treatment after release (World Health Organization, 2014b). Previous studies have found positive effects of in-prison DUD treatment, including Opioid Agonist Treatment (OAT) (European Monitoring Centre for Drugs and Drug Addiction, 2022; World Health Organization, 2014a, 2014b), regarding reductions in drug use (Andrade et al., 2018; Doyle et al., 2019; Moore et al., 2019), mortality (including overdose deaths) (Bukten & Stavseth, 2024; Degenhardt et al., 2014, 2019; European Monitoring Centre for Drugs and Drug Addiction, 2022; Lim et al., 2023; World Health Organization, 2014a), and return to prison (Andrade et al., 2018; Gisev et al., 2019). A recent Norwegian study on OAT in prison, found a reduction in both all-cause mortality (hazard ratio = 0.58, 95% CI 0.39–0.85) and overdose mortality (HR = 0.51, 0.31–0.82), among people with opioid use disorder who continued treatment during imprisonment (Bukten & Stavseth, 2024).

According to the internationally recognized principles of equivalence of care and continuity of care, people in prison should have access to the same standards of health care that are available in the community, without discrimination on the grounds of their legal status (European Monitoring Centre for Drugs and Drug Addiction, 2022; Office of the High Commisioner for Human Rights & World Health Organization, 2008). Though the availability of drug use disorder treatment within the prison setting has improved in the last twenty years (European Monitoring Centre for Drugs and Drug Addiction, 2022), the actual treatment coverage in prisons remains unexplored in most settings. A recent nationwide cohort study from Denmark, found that 34.6% of incarcerated people with a history of substance use disorder received treatment during imprisonment (Seid et al., 2024). People with a history of poly-drug problems most often received treatment (52.4%) as opposed to people with alcohol use disorder (27.5%), which was also the most common SUD in this sample (Seid et al., 2024). In Norway, Bukten et al. (2023) found an increase in OAT coverage for people with opioid use disorder, from 36% in 2010 to 70% in 2019 (Bukten et al., 2023).

However, though research based on national registry data have great methodological advantages, patient and treatment data will often underestimate the treatment need in the population.

The actual need for drug use disorder treatment among people in prison, can be difficult to estimate if people entering prisons are not systematically screened, which is the main reason why the treatment coverage across most European countries remains unknown (European Monitoring Centre for Drugs and Drug Addiction, 2022). To estimate treatment coverage and underserved groups within the prison population, we need individual level data from a representative sample of the prison population on drug use and self-perceived treatment needs as well as complete data on drug use disorder treatment utilization during imprisonment. Furthermore, to facilitate treatment and interventions, health policy and health care provision must be based on knowledge of the specific needs of the local population.

## Aims

The overall aims of this study were to describe treatment needs before incarceration and utilization of drug use disorder treatment during imprisonment using data from a national longitudinal prison cohort.

The specific objectives of our analyses were to:

1. Estimate the need for drug use disorder treatment in Norwegian prisons and describe the characteristics of people with drug use disorder treatment needs.
2. Estimate utilization of DRUG USE DISORDER treatment in prison and the characteristics of those who receive treatment; and.
3. Investigate sociodemographic and imprisonment factors associated with utilization of DRUG USE DISORDER treatment during imprisonment, among people with need for DRUG USE DISORDER treatment.

## Materials and methods

### Setting

Norway has one of the lowest imprisonment rates in the world, with an average of 3,218 individuals imprisoned in 2019, equal to an imprisonment rate of 60 per 100,000 of the national population (The Directorate of the Norwegian Correctional Service, 2020). Almost two-thirds of prisons are high security prisons. The yearly turnover is high, as 85% of prison sentences are less than one year, 50% serve less than three months and most prisoners with sentences longer than 74 days are released on parole after two-thirds time served (The Directorate of the Norwegian Correctional Service, 2018).

Women constitute a minority in Norwegian prisons, with an annual proportion of approximately 6%. Norway has one of the lowest imprisonment rates in the world, with an average of 3,218 individuals imprisoned in 2019, equal to an imprisonment rate of 60 per 100,000 of the national population (The Directorate of the Norwegian Correctional Service, 2020). Almost two-thirds of prisons are high security prisons. The yearly turnover is high, as 85% of prison sentences are less than one year, 50% serve less than three months and most prisoners with sentences longer than 74 days are released on parole after two-thirds time served (The Directorate of the Norwegian Correctional Service, 2018). Women constitute a minority in Norwegian prisons, with an annual proportion of approximately 6%.

Norway is characterized by universal health coverage, and people in prison thus retain all rights to access health care during imprisonment. Health care services in prison are delivered by the health care providers from the community where the prison is located, with primary health care services organized by the municipality and specialized health care services, including DRUG USE DISORDER treatment, organized by the regional health authorities (Fridhov & Langelid, 2017; The Norwegian Directorate of Health, 2016). People with drug use disorder in prison can access treatment from health care providers inside and outside the prison or apply for treatment at Drug Counselling Units in collaboration between correctional services and the specialized health care services (Helgesen, 2019). People diagnosed with opioid use disorder have the opportunity to continue or enter OAT in prison, while Norwegian legislation also allows for people with drug use disorder to serve all or part of a sentence in an inpatient treatment facility outside the prison, provided by the specialized health care services (The Norwegian Directorate of Health, 2016). However, despite the universal rights and treatment facilities, there is reason to believe that both access to and the quality of the treatment delivered in prison, differ from treatment provided on the outside (European Monitoring Centre for Drugs and Drug Addiction, 2022; Oslo Economics & Tyrilsstiftelsen, 2020; Ugelvik & Dullum, 2012).

### Design

This study is a longitudinal cohort study using data from the Norwegian Mental Health and Addiction (NorMA) cohort (*n* = 733) collected in 2013-14, during visits to 57 prison units (of 63 available) throughout Norway (Bukten et al., 2015). Data collection took place in both high- and low-security units and transitional houses, including the three all-women prisons in Norway (Bukten et al., 2015). A one-page consent form was included in the beginning of the questionnaire, explaining the purpose of the study and the confidentiality of the answers. Furthermore, participants were asked to enroll in the cohort study, by providing their personal identification number, and their consent to its use for linking the survey responses with registry data. People were encouraged to contribute to the cross-sectional part of the study, even if they could not or would not provide personal identification number (Bukten et al., 2015).

The baseline imprisonment was defined as the imprisonment in which the participants responded to the NorMA survey. For a thorough description of the methodology and study design, see Bukten et al. (2015a, b) (Bukten, Lund, Rognli, Bukten et al., 2015a, b).

### Data sources

Data from the NorMA cohort included baseline survey data on mental health and substance use, as well as demographics and background information. The baseline survey data was linked to the Norwegian Patient Registry (2009–2019) and the Norwegian Prison Registry (2000–2019) by using the PIN (Bukten et al., 2015). The registry data made it possible to obtain information about the NorMA cohort participants before, during, and after their index imprisonment. The index imprisonment was defined as the ongoing imprisonment at the time of inclusion into the study.

The Norwegian Prison Registry is administered by the Norwegian Correctional Service and was established in 1992. The registry includes data from all Norwegian prisons on sentences, imprisonment status, security level and participation in programs (Bukten et al., 2015).

The Norwegian Patient Registry (NPR) was established in 2009 as a national registry covering all patients receiving specialized health care. This includes treatment received in government-owned hospitals and clinics, and private clinics with governmental reimbursement.

### Participants

The questionnaire was administered by the study investigators and distributed to the participants on the day of the data collection visit (Bukten et al., 2015). The only criteria for inclusion were the willingness and ability to complete the NorMA questionnaire and to provide a Norwegian personal identification number, which includes foreign nationals with a right to access public welfare services but excludes people with foreign nationality without a legal residence permit. All inmates imprisoned in Norway at the time of data collection were encouraged to participate regardless of nationality, age, gender, or health status, and the questionnaire was available in Norwegian, English, Russian, French, and German (Bukten et al., 2015). A total of 1,495 people returned the NorMA questionnaire and 733 provided a personal identification number, henceforth constituting the NorMA cohort. A previous study investigated potential selection into the NorMA cohort and found that the NorMA cohort was representative of the Norwegian prison population in possession of a Norwegian personal identification number (Toresen Lokdam et al., 2021).

### Measures

The measures in the descriptive analysis included data from both the NorMA-survey and registry data. A full description of all measures is included in the supplementary material (Supplementary Table 1).

Treatment need was estimated with the Drug Use Disorder Identification Test (DUDIT) which has previously been validated in a prison setting (Berman et al., 2005; Durbeej et al., 2010; Pape et al., 2022). The 11-item instrument measured self-reported drug use and drug use behavior in the year before imprisonment, with a sum score ranging from 0 to 44. A score ≥ 6 is indicative of harmful drug use (i.e., full assessment and evaluation recommended), while a score ≥ 25 indicates a likely drug dependence (Berman et al., 2005). Based on these cutoffs, we categorized persons as having ‘low-risk drug use’ (< 6), ‘harmful drug use’ (6–24) or ‘likely drug dependence’ (≥ 25). As some scores defined as harmful were close to the cutoff for ‘likely drug dependence’, we included all people categorized with harmful drug use or likely drug dependence, when estimating factors associated with drug use disorder treatment utilization. Validation studies of the DUDIT have recommend adjusted cutoff scores for women (Basedow et al., 2021; Durbeej et al., 2010; Hildebrand, 2015). However, we used the same cutoffs for all, as previous research on the full NorMA sample found similar patterns of drug use among men and women (Bukten et al., 2020; Pape et al., 2020).

The main outcome was the utilization of drug use disorder treatment during baseline incarceration, defined as at least one treatment episode registered with the diagnostic codes F11-F19 of the ICD-10. Treatment utilization in the year before imprisonment was included as a covariate in the descriptive analysis. F11-F19 contain a wide variety of mental and behavioral disorders that are all attributable to the use of one or more psychoactive substance. The specific codes refer to the use of F11: Opioids, F12: Cannabinoids, F13: Sedatives or hypnotics, F14: Cocaine, F15: Other stimulants, F16: Hallucinogen, F17: Nicotine, F18: Inhalants, and F19: Multiple drug use and use of other psychoactive substances. Due to the limited prevalence of F16, F17 and F18 (*n* ≤ 1) these three diagnoses were excluded from our analysis.

Demographic variables included: Gender, age, country of birth (Nordic born/non-Nordic), education (primary school level or less/more than primary school), occupational status (employed or enrolled in education/no employment) and accommodation before imprisonment (stable/unstable housing). Social problems in upbringing were measured as life-time experiences with being placed in foster care (yes/no) and growing up with parents having problems with psychiatric illness and/or substance use (yes/no). Symptoms of mental distress (depression/anxiety) was measured with the Hopkins symptom checklist (HSCL-10) (Derogatis et al., 1974). Additional measures of drug use included self-reported frequency of injecting drug use and weekly polydrug use in the last 6 months before imprisonment. Treatment motivation was measured with the DUDIT-Extended (DUDIT-E) (Berman et al., 2007). We also included information on previous imprisonments, drug use related crime and length of baseline imprisonment.

### Statistical analysis

Statistical analyses were performed in Stata (Version 16). We performed descriptive statistics on complete case data from the survey, the prison registry, and the Norwegian Patient Registry. First, demographic and imprisonment characteristics were reported for all cases stratified for DUDIT category (‘low-risk drug use’, ‘harmful drug use’, and ‘likely drug dependence’). Second, characteristics related to demography, drug use, mental health, imprisonment, and DUD treatment the year before imprisonment, were reported according to treatment status (DUD treatment during baseline: yes/no). Third, the association between DUD treatment status and relevant factors among people with harmful drug use or likely drug dependence was investigated using a logistic regression model. Unadjusted odds ratios (OR) from univariate models and adjusted odds ratios (aOR) were reported with corresponding confidence intervals (CI’s).

The level of missing data in the baseline material ranged from 0 to 56% (DUDIT-E: Treatment motivation) with 289 complete cases (39%). Our exposure variable, the sum score of all DUDIT items, had 11% missing. A detailed list of missing data is shown in Table 1. As we did not consider the data as missing completely at random (MCAR), we pre-processed the data before running the regression analysis by imputing all variables with missing data using Multiple Imputation by Chained Equations (MICE). In line with the ‘Treatment and Reporting of Missing Data in Observational Studies’-framework by Lee et al. (2021) our imputation model included the variables from our regression model (Lee et al., 2021): exposure, outcome and potential confounders (sex, age, education, length of imprisonment, injecting drug use before imprisonment and poly-drug use). MICE was conducted using ‘mi impute’ with 100 imputations and 1000 iterations. The regression coefficients were pooled using the Stata function ‘mi estimate’ based on Rubin’s rules (Rubin, 1987). The full regression model was constructed using a directed acyclic graph (DAG), as illustrated in supplementary material. The regression model included all covariates identified as confounder in the DAG, except for gender, because it was statistically insignificant and therefore removed from the final model. Removing gender from the model had no influence on neither estimates nor p-values. The full model, including gender, can be seen in the supplementary material (Supplementary Table 2), which also contain more information on diagnostics, sensitivity analysis of the MICE model and the DAG.

**Table 1:** Descriptive characteristics, by DUDIT category

Self-reported drug use: DUDIT category*	Low-risk drug use	Harmful drug use	Likely drug dependence	Total	Missing
	238 (32.5)	119 (16.2)	294 (40.1)	651 (89.0)	82 (11.2)
**Sociodemographic**					
Gender (male)	220 (92.4)	112 (94.1)	274 (93.2)	606 (93.1)	0 (0.0)
Age, mean (SD)	39.5 (13.4)	32.1 (10.1)	33.5 (8.8)	35.5 (11.6)	0 (0.0)
Nordic born	184 (77.3)	103 (86.6)	265 (90.1)	552 (84.8)	18 (2.8)
Education: More than primary school	170 (71.4)	60 (50.4)	145 (49.3)	375 (57.6)	9 (1.4)
Occupation: Job or education	156 (65.5)	53 (44.5)	55 (18.7)	264 (40.6)	16 (2.5)
Accommodation: Stable housing	205 (86.1)	93 (78.2)	173 (58.8)	471 (72.4)	26 (4.0)
Foster care	33 (13.9)	24 (20.2)	72 (24.5)	129 (19.8)	16 (2.5)
Problems in childhood	49 (20.6)	40 (33.6)	136 (46.3)	225 (34.6)	26 (4.0)
**Health and drug use**					
HSCL-10 score ≥ 1.85	62 (26.1)	37 (31.1)	140 (47.6)	239 (36.7)	164 (25.2)
Injecting drug use last 6 months					53 (8.1)
No injecting drug use	233 (97.9)	90 (75.6)	101 (34.4)	424 (65.1)	
Daily/almost daily	0 (0.0)	7 (5.9)	146 (49.7)	153 (23.5)	
1–2 times per week	0 (0.0)	8 (6.7)	25 (8.5)	33 (5.1)	
1–3 times per month	0 (0.0)	11 (9.2)	9 (3.1)	20 (3.1)	
Polydrug use (weekly)	0 (0.0)	29 (24.4)	245 (83.3)	274 (42.1)	41 (6.3)
**Treatment motivation**					361 (55.5)
Low	83 (34.9)	47 (39.5)	104 (35.4)	234 (35.9)	
Middle	4 (1.7)	12 (10.1)	66 (22.4)	82 (12.6)	
High	4 (1.7)	4 (3.4)	25 (8.5)	33 (5.1)	
**DUD treatment**					
Year before imprisonment	5 (2.1)	25 (21.0)	160 (54.4)	190 (29.2)	0 (0.0)
During baseline imprisonment	6 (2.5)	29 (24.4)	187 (63.6)	256 (34.1)	0 (0.0)
**Imprisonment**					
Previous imprisoned	108 (45.4)	88 (73.9)	260 (88.4)	456 (70.0)	0 (0.0)
Median number of previous imprisonment (Q1-Q3)	0 (0–1)	2 (2–3)	4 (3–4)	2 (1–3)	0 (0.0)
Any drug use-related convictions	31 (13.0)	55 (46.2)	186 (63.3)	272 (41.8)	0 (0.0)
**Length of imprisonment**					0 (0.0)
< 3 months	54 (22.7)	30 (25.2)	38 (12.9)	122 (18.7)	
3–6 months	26 (10.9)	18 (15.1)	39 (13.3)	83 (12.7)	
6–12 months	28 (11.8)	18 (15.1)	88 (29.9)	134 (20.6)	
> 12 months	128 (53.8)	53 (44.5)	129 (43.9)	310 (47.6)	

Demographic and imprisonment characteristics (n, %), by reported DUDIT score, total and missing (*n* = 733). Percentages are calculated among all cases, including missing, and may therefore not add up to 100%. *The DUDIT has a sum score range from 0–44. The DUDIT categories are ‘low-risk use’ (< 6), ‘harmful use’ (6–24) and ‘likely drug dependence’ (≥ 25). First row presents *n* and % for each DUDIT category, total and missing among the total NorMA cohort (*n* = 733)

## Results

### Estimating the need for DUD treatment

The NorMA cohort (*n* = 733) included 45 (7%) women, and the mean age of all participants at baseline was 35.5 years (SD = 11.6). When answering the DUDIT, 238 (33%) reported low-risk, 119 (16%) reported harmful drug use and 294 (40%) likely drug dependence before imprisonment (Table 1). Compared to persons reporting low-risk and harmful use, persons with likely dependence reported more socio-demographic problems such as unstable housing, less education, and more severe drug use, characterized by weekly polydrug use (83%) and daily/almost daily injecting drug use (50%) in the six months leading up to their imprisonment.

In the year before imprisonment, 54% of people with likely dependence and 21% of people with harmful drug use had received DUD treatment. During baseline imprisonment, 64% of people with likely drug dependence and 24% of people with harmful drug use received DUD treatment.

46% of people reporting harmful use and 63% of people with likely dependence had a drug use-related conviction. In all three groups, a sentence of more than 1 year was most common (low-risk: 54%, harmful use: 44%, likely dependence: 44%).

### Characteristics of people who received drug use disorder treatment in prison

Among the NorMA cohort (*n* = 733), 256 people (35%) had received drug use disorder treatment during their baseline imprisonment (Table 2). Compared to the no treatment group, those who received drug use disorder treatment were younger (mean age, treatment: 33 years vs. no treatment: 37 years) and more often Nordic born (93% vs. 79%), and fewer had more than primary school (46% vs. 64%), occupation before imprisonment (22% vs. 51%) or stable housing (61% vs. 78%). People receiving treatment had more social problems during upbringing, including experiences with foster care (treatment group: 27% vs. no treatment group: 17%) and parental substance use or psychiatric illness (47% vs. 30%). People who received treatment during imprisonment had more previous imprisonments (median four versus one in the no-treatment group), drug use related crime (66% vs. 30%) and longer baseline imprisonment (median: 12 vs. 10 months).

**Table 2:** Descriptive characteristics, by DUD treatment status (*n* = 733)

	No DUD treatment		Any DUD treatment		Total	Missing	
	477 (65.1)		256 (34.9)		733 (100)	0 (0.0)	
**Demographics**							
Female	37 (7.8)		14 (5.5)		51 (7.0)	0 (0.0)	
Age, mean (SD)	37.0 (12.8)		32.8 (8.3)		35.5 (11.6)	0 (0.0)	
Nordic born	375 (78.6)		237 (92.6)		612 (83.5)	18 (2.5)	
Education: More than primary school	307 (64.4)		117 (45.7)		424 (57.8)	9 (1.2)	
Occupation: Job or education	243 (50.9)		55 (21.5)		298 (40.7)	16 (2.2)	
Problems in childhood: Yes	144 (30.2)		121 (47.3)		265 (36.2)	26 (3.5)	
Foster care: Yes	80 (16.8)		70 (27.3)		150 (20.5)	16 (2.2)	
Accommodation: Stable housing	370 (77.6)		156 (60.9)		526 (71.8)	26 (3.5)	
**Imprisonment**							
Previously imprisoned	284 (59.5)		232 (90.6)		516 (70.4)	0 (0.0)	
Median number of previous imprisonment (Q1-Q3)	1 (1–1)		4 (4–5)		2 (2–2)	1 (0.1)	
Drug use-related conviction	141 (29.6)		170 (66.4)		311 (42.4)	0 (0.0)	
**Length of imprisonment**						0 (0.0)	
< 3 months	122 (25.6)		15 (5.9)		137 (18.7)		
3–6 months	65 (13.6)		31 (12.1)		96 (13.1)		
6–12 months	76 (15.9)		81 (31.6)		155 (21.1)		
12 < months	214 (44.9)		129 (50.4)		343 (46.8)		
Median (Q1-Q3)	9.7 (7.9–12.0)		12.2 (10.6–14.3)		11.0 (9.9–12.3)		
**Drug use**							
Level of drug use 1 year before imprisonment						82 (11.2)	
Low risk	232 (48.6)		6 (2.3)		238 (32.5)		
Harmful	90 (18.9)		29 (11.3)		119 (16.2)		
Likely drug dependence	107 (22.4)		187 (73.0)		294 (40.1)		
Weekly drug use^1^	185 (38.8)		226 (88.3)		411 (56.1)	41 (5.6)	
Weekly polydrug use^1^	113 (23.7)		189 (73.8)		302 (41.2)	41 (5.6)	
Injecting drug use (IDU)^1^						53 (7.2)	
No IDU	369 (77.4)		87 (34.0)		456 (62.2)		
Daily/almost daily	51 (10.7)		114 (44.5)		165 (22.5)		
1–2 times per week	14 (2.9)		21 (8.2)		35 (4.8)		
1–3 times per month	11 (2.3)		13 (5.1)		24 (3.3)		
**Mental health and treatment**							
Treatment motivation						361 (49.2)	
Low	166 (34.8)		81 (31.6)		247 (33.7)		
Middle	32 (6.7)		56 (21.9)		88 (12.0)		
High	15 (3.1)		22 (8.6)		37 (5.0)		
Severe mental distress: HSCL-10 score > 1.85	141 (29.6)		115 (44.9)		256 (34.9)	164 (22.4)	
DUD Treatment the year before imprisonment^2^							
No treatment	416 (87.2)		95 (37.1)		511 (69.7)	0 (0.0)	
Any treatment	61 (12.8)		161 (62.9)		222 (30.3)	0 (0.0)	

Characteristics of demography, imprisonment, drug use, mental health and treatment utilization (n, %) by DUD treatment status, total and missing for all participants (*n* = 733). Percentages are calculated among all cases, including missing, and may therefore not add up to 100%. ^1^Drug use 6 months before imprisonment, ^2^Any treatment related to F11-19 in the year leading up to imprisonment (missing = 0)

Among people receiving treatment during baseline imprisonment, 73% reported likely drug dependence, 88% reported using drugs daily or almost daily, 74% reported weekly polydrug use and 45% reported daily/almost daily injecting drug use. Most people in the treatment group reported low (32%) or middle (22%) treatment motivation. Among people who received treatment during imprisonment, 161 (63%) had also received treatment in the year leading up to their imprisonment.

### Factors associated with utilization of DUD treatment during imprisonment

Several factors were associated with drug use disorder treatment (Table 3). Longer imprisonment increased the chances of receiving treatment during imprisonment. People imprisoned for one year or more were most likely to receive DUD treatment during imprisonment (aOR = 8.87, *p* < 0.001) compared to people imprisoned for less than three months. People born in a Nordic country had almost three times higher odds of receiving treatment during imprisonment, compared to people born outside the Nordics (aOR = 2.85, *p* = 0.003). Polydrug use (aOR = 2.19, *p* = 0.002) and daily injecting drug use (aOR = 2.58, *p* < 0.001) increased the chances of receiving treatment.

**Table 3:** Logistic regression model on treatment status

Outcome: Treatment (any treatment /no treatment)	Univariate		Adjusted	
	OR (95% CI)	*p*	aOR (95% CI)	*p*
Nordic born	**2.96 (1.58–5.56)**	**0.001**	**2.85 (1.42–5.73)**	**0.003**
Education: More than primary school	**0.65 (0.45–0.93)**	**0.018**	0.73 (0.48–1.11)	0.140
Age at baseline	0.99 (0.97–1.01)	0.248	0.98 (0.96-1.00)	0.103
Length of imprisonment				
3–6	**3.46 (1.65–7.25)**	**0.001**	**3.44 (1.58–7.52)**	**0.002**
6–12	**7.02 (3.60-13.68)**	**< 0.001**	**6.33 (3.11–12.89)**	**< 0.001**
> 12	**6.40 (3.42–11.97)**	**< 0.001**	**8.87 (4.48–17.55)**	**< 0.001**
IDU				
Daily/almost daily	**3.41 (2.23–5.21)**	**< 0.001**	**2.58 (1.51–4.39)**	**< 0.001**
1–2 per week	**2.40 (1.17–4.95)**	**0.017**	2.24 (0.96–5.23)	0.063
1–3 per month	1.93 (0.83–4.49)	0.126	2.06 (0.79–5.38)	0.138
Polydrug use	**3.56 (2.36–5.36)**	**< 0.001**	**2.19 (1.34–3.60)**	**0.002**
Cons.			0.07 (0.02–0.22)	< 0.001

Logistic regression model estimates based on pooled, imputed data, giving unadjusted and adjusted odds ratios (OR and aOR), 95% confidence intervals (CI) and p-values. Sample of cohort participants with harmful drug use or likely dependence before imprisonment (*n* = 483)

## Discussion

By using a combination of high-quality survey and national registry data, this study contributes to filling the knowledge gap on coverage of drug use disorder treatment in prisons. People entering prison with likely dependence, was a group characterized by social marginalization, high prevalence of polydrug use and injecting drug use, and previous imprisonments, often with drug use related convictions.

Our results showed that 40% of the population had been likely drug dependent when entering prison and that more than 60% of the likely drug dependent received drug use disorder treatment during baseline imprisonment.

Our findings resemble the 66–69% treatment coverage estimated by Degenhardt et al. (2017) for the general population in high-income countries (Degenhardt et al., 2017). They also estimated that only 10% of people with SUD received “minimally adequate treatment”. “Minimally adequate treatment” was defined as receiving 4 sessions from specialized mental health or general medical provider, or 6 from non-medically trained professionals (Degenhardt et al., 2017). As our study did not assess the quantity and quality of the treatment provided, we can therefore conclude whether the treatment provided was adequate or not.

People who received treatment in prison had a heavier burden of social and economic problems and were younger, male, and born in a Nordic country. Of those who received drug use disorder treatment before imprisonment, more than 70% received treatment during imprisonment.

Furthermore, 19% of those who had not received treatment in the year before imprisonment, did so in prison and people with likely drug dependence had the highest increase in treatment during imprisonment. However, 30% of people who were likely dependent did not receive treatment during imprisonment, which indicates a treatment gap. Similarly, Seid et al. (2024), found a coverage of 34.6% among people who were previously diagnosed before entering prison, but higher coverage among people with disorders related to opioid, cocaine, amphetamine or polysubstance use, while people diagnosed with alcohol use disorder were less likely to receive treatment. These findings could indicate that people with more complex substance use disorders experience a better treatment coverage when entering prison, compared to people with less severe substance use disorders in prison.

People who spent more than three months in prison, especially people imprisoned for one year or more, were more likely to receive DUD treatment during imprisonment. The association with sentence length has also been found in other studies in the Scandinavian context, with similar sentence lengths (Seid et al., 2024). This increased chance of accessing treatment could be attributed to the delay in referral and treatment of drug use disorder in prison, which corresponds to a general delay in substance use disorder treatment. In 2015, the median waiting time for specialized drug use disorder treatment in Norway was 31 days (Fuglset et al., 2016). The delay could also be explained by factors related to the prison setting and environment, as the individual might feel a need to build up trust in the health care providers within the prison before entering treatment. Furthermore, depending on the drug use disorder diagnosis and type of treatment, the patient and health care provider might choose to initiate treatment after release, especially when the sentences are short. However, as 40% of people with harmful use and 26% of people with likely drug dependence spend less than six months in prison, the screening, referral and treatment systems should be adapted to accommodate those with sentences shorter than six months.

People born in the Nordics had three times higher odds of receiving treatment, compared to people born outside the Nordics. This could indicate a barrier in assessment of treatment needs or access to health care services for those with a non-Nordic background. Some of this association might be explained by residual confounding, as fewer people born outside the Nordics had likely drug dependence. In Denmark, Seid et al. (2024), found that immigrants were less likely to receive prison-based substance use disorder treatment (OR = 0.92, 95% CI 0.86–0.99), while children of immigrants were more likely to receive treatment, compared to native Danish individuals (OR = 1.18, 1.08–1.30). The authors suggest these differences can be related to both language barriers, but also that some immigrants, who are not Danish citizens and could therefore be convicted to deportation, would not be entitled to receive this kind of treatment while in prison according to Danish law (Seid et al., 2024). Such legal barriers could also be present in the Norwegian context, as Norway have a somewhat similar legislation. Furthermore, some of these differences could be explained by cultural differences in drug use patterns, if they use different drugs or have different treatment needs. For instance, Bukten et al.(2023) found a lower prevalence of opioid use disorder among immigrants, compared to Norwegian born people in prison (Bukten et al., 2023). However, further analysis of the characteristics of this group was unfeasible, due to small sample sizes. This association therefor remains unexplained and should be studied further.

In addition, there can be other explanations why people do not receive treatment during their imprisonment, including reasons related to the individual or the prison environment. Hence, people could be hesitant to share their history of substance use with staff in the prison, out of fear for disciplinary sanctions or other negative consequences, either from the correctional services or from other people imprisonned (Kolind et al., 2010).

### Strength and limitations

By using survey data in combination with registry data, we had the opportunity to look at the relationship between the need for treatment and actual treatment involvement at an individual level. Having access to self-reported data made it possible to identify levels of pre-prison drug use according to the DUDIT; a standardized tool, validated for use in the prison setting (Berman et al., 2005; Pape et al., 2022). In addition, rich survey data enabled us to adjust for a wide range of background characteristics that is not obtained in the registries. The registry data from the Norwegian Prison Registry and the Norwegian Patient Registry provided complete information about the imprisonment and the utilization of treatment during imprisonment.

However, this study has some limitations, which should be taken into consideration. First, we studied the utilization of drug use disorder treatment as a simple binary outcome (treatment/no treatment). This would include drug use disorder treatments of a varied nature, from acute intoxications to opioid agonist treatment and does not give detailed information on the intensity or content of the treatment, nor to what degree the treatment adequately covered the treatment needs of the person. Second, the Norwegian Patient Registry did not include information about treatment provided by other health care providers than the specialized health care services, e.g. from primary health care providers in the municipalities. This could lead to an underestimation of treatment coverage. Third, future research should investigate if people continue or begin treatment after release or remain out of treatment upon release. Fourth, complex questions regarding treatment motivation and drug use patterns may vary depending on time and place and can be challenging to measure in a cross-sectional survey. Hence, recall bias and social desirability bias are always an issue in self-reported data and may have led to underreporting of pre-prison drug use. On the other hand, for persons receiving treatment in prison, increased awareness of substance related problems could affect reporting of own substance use prior to prison, which could possibly inflate the association between drug use and treatment utilization. Finally, we did not study psychiatric comorbidity and some people with likely drug dependence might have received treatment for other psychiatric disorders, or abstinence related symptoms, which could have reduced the need for drug use disorder treatment.

### Implications

In-prison treatment for drug use disorders is effective for reducing reimprisonment, and post-release mortality (Bukten & Stavseth, 2024; Andrade et al., 2018; Degenhardt et al., 2014, 2019; Doyle et al., 2019; European Monitoring Centre for Drugs and Drug Addiction, 2022; Gisev et al., 2019; Lim et al., 2023; Moore et al., 2019; World Health Organization, 2014a). Thus, information on treatment coverage is fundamental for both policy makers and clinicians to facilitate the best possible treatment in prison. However, both globally and in Norway, the actual treatment coverage for people with drug use disorders in prison has remained unknown. To estimate coverage, it is essential to perform systematic screenings upon entry to prison, to identify the treatment needs. Screenings should be based on standardized tools, such as the DUDIT, or its brief versions, which have been shown to perform well detecting likely dependence in the prison population (Pape et al., 2022).

The universal right to health for people in prisons implies that health care providers are obliged to make treatment accessible for all people in need of treatment. People in prison must have access to suitable assessment and treatment, and it may be necessary to implement additional interventions targeting their specific needs (European Monitoring Centre for Drugs and Drug Addiction, 2022; World Health Organization, 2014b). Further studies should investigate the possible individual or structural barriers resulting in treatment gaps for people with short sentences and other nationalities.

The qualities of Norwegian prison system have, together with the other Scandinavian countries, often been characterized as Scandinavian exceptionalism, representing particular humane prison standards, imbedded in the Scandinavian Welfare states. However, the Norwegian prison population also represent a population with an increasing burden of health challenges, with a high need for treatment and health care provision. Though Norway has seen an increasing treatment coverage for some substance use disorders, in particular for people with opioid use disorder in OAT (Bukten & Stavseth, 2024), there remain treatment gaps both for people with opioid use disorder and for people with others SUDs, e.g. alcohol use disorder. Future research should investigate the treatment gaps across primary and specialized health care providers, and potential benefits of improving substance use disorder treatment, also for people using other substances other than opioids.

Some of the challenges of providing health care that were identified in our study, might therefore also apply to other settings. Norway represents a country with universal health rights for people in prison, in combination with a national patient registry available for research. This entails an important opportunity for conducting research on health care provision for people in prison, to inform and guide the development of health care services in other national settings. In that regard, the great diversity in national prison contexts, makes it important to study the health challenges and treatment provision across different national context, being able to inform local policy with country-specific context and research.

## Conclusion

Our results from a national prison cohort show that more than 60% of those incarcerated with likely drug dependence access DUD treatment during their imprisonment. While characteristics of severe drug use were strongly associated with more treatment utilization in this study, the length of imprisonment and country of birth were associated with less treatment utilization. Our findings point to barriers to the provision of DUD treatment for people in Norwegian prisons that should be addressed to further reduce the treatment gap among people with drug use disorders in prison.

## Electronic supplementary material

Below is the link to the electronic supplementary material.

Supplementary Material 1

## Data Availability

The data that support the findings of this study are highly sensitive and were used under ethical approvals for the PriSUD project, and so are not publicly available. Questions regarding the data can be forwarded to co-author Anne Bukten, PI of the PriSUD project.
