# Supplementary material for: Treatment utilization among people with drug use disorders in prison: a national longitudinal cohort study
Source: Health Justice. 2024 Nov 26;12:46. doi: 10.1186/s40352-024-00302-8 (PMC11590615; doi:10.1186/s40352-024-00302-8)
Supplement: Supplementary file 1 — Supplementary Material 1 [file 40352_2024_302_MOESM1_ESM.docx]

**Supplementary table 1:** Details on all measures in the descriptive analysis, describing their source and content.

| **Variable** | **Source** | **Description** |
| --- | --- | --- |
| **Demographics** |  |  |
| Gender | Survey | Female vs. male |
| Age | Registry | Continues variable |
| Nordic | Survey | Born in a Nordic country vs. outside the Nordic countries |
| **Socioeconomics** |  |  |
| Education | Survey | More than primary school vs. only primary school or less. |
| Occupation | Survey | Part-time or full-time job or education before baseline imprisonment vs. no occupation |
| Problems in childhood | Survey | Being in a family with drug use and/or psychiatric disorders in childhood vs no problems |
| Foster care | Survey | Any experience with foster care at any time during childhood vs. only growing up with biological parents. |
| Accommodation | Survey | Unstable housing situation before imprisonment vs. stable housing. |
| **Health and drug use** |  |  |
| HSCL-10 score ≥1.85 | Survey | Hopkins symptom check list (HSCL-10), measuring symptoms of psychological distress (Derogatis, Lipman, Rickels, Uhlenhuth, & Covi, 1974). Scores ≥18.5 indicate clinical concern. |
| IDU | Survey | Status of injecting drug use last 6 months before imprisonment. Categories: No IDU, Daily/almost daily, 1-2 times per week or 1-3 times per month. |
| Polydrug use | Survey | Weekly use of more than two substances (not including alcohol) during the 6 months leading up to baseline imprisonment vs. no polydrug use. |
| Level of drug use | Survey | Drug Use Disorder Identification Test (DUDIT). 11 items instrument to map the frequency of substance use in the year leading up to incarceration. Scores from 0-44. Categorized as low-risk (<6), harmful substance use (6-24) and likely drug dependence (>24). |
| Treatment motivation | Survey | Drug Use Disorder Identification Test – Extended (DUDIT-E). Instrument measuring perceived positive aspects of using drugs (17 items), negative aspects (17 items) and treatment readiness (10 items). Used as a combined motivation index according to Berman et al (2007) (Berman, Palmstierna, Kallmen, & Bergman, 2007) with three categories: low, middle, and high motivation. |
| **Imprisonment** |  |  |
| Previous imprisoned | Registry | Any imprisonments before baseline vs. none. |
| Previous imprisonments | Registry | Median number of imprisonments before baseline. |
| Any drug use-related crime | Registry | Substance use related crime among baseline convictions, defined as “use and possession” and/or “intoxicated driving”, yes/no. |
| Length of imprisonment | Registry | Length of baseline imprisonment shown as median and in four categories: Less than 3 months, 3-6 months, 6-12 months, and 12 months or more. |

SENSITIVITY ANALYSIS

We performed diagnostics of the imputation model were, comparing the chosen model to complete case analysis and an extended imputation model with additional auxiliary variables. First, we compared the imputed values by visual inspection with observed values in all imputations. We also assessed the percentage of persons assigned to each exposure group (low-risk use/harmful use/likely drug dependence according to the DUDIT). In addition, we conducted two sets of sensitivity analyses to examine the effect of changes to the imputation model on the estimates from the regression analysis.

The sensitivity analysis were conducted to investigate the effect of including additional auxiliary variables in the imputation model, compared to full model and complete case analysis. Based on the sensitivity analysis, we chose an imputation model equal to the regression analysis (Nordic born, ‘education’, IDU and polydrug use. We included age, sex and length of imprisonment as auxiliary variables since these had no missing data.

**Sensitivity analysis A:** Logistic regression analysis on complete case dataset compared to dataset imputed with a full model (all variables from the logistic regression model) and a full model with additional auxiliary variables. The estimates are shown as adjusted odds ratios (aOR) with 95% confidence intervals (CI) and p-value.

| **Any DUD treatment during imprisonment** | **Complete case (n=383)** | | **Full model (n=483)** | | **Full model + auxiliary variables* (n=470)** | |
| --- | --- | --- | --- | --- | --- | --- |
|  | **aOR (95% CI)** | ***P*** | **aOR (95% CI)** | ***P*** | **aOR (95% CI)** | ***P*** |
| Nordic born | 2.71 (1.18-6.21) | 0.019 | 2.85 (1.42-5.73) | 0.003 | 2.64 (1.30-5.37) | 0.007 |
| Education: More than primary school | 0.73 (0.46-1.17) | 0.197 | 0.73 (0.48-1.11) | 0.140 | 0.75 (0.49-1.14) | 0.177 |
| Age at baseline | 0.98 (0.96-1.01) | 0.223 | 0.98 (0.96-1.00) | 0.103 | 0.99 (0.96-1.01) | 0.228 |
| Length of imprisonment |  |  |  |  |  |  |
| 3> (ref.) |  |  |  |  |  |  |
| 3-6 | 3.60 (1.49-8.68) | 0.004 | 3.44 (1.58-7.52) | 0.002 | 3.38 (1.55-7.40) | 0.002 |
| 6-12 | 7.96 (3.50-18-13) | <0.001 | 6.33 (3.11-12.89) | <0.001 | 6.27 (3.07-12.80) | <0.001 |
| 12< | 11.10 (5.05-24.37) | <0.001 | 8.87 (4.48-17.55) | <0.001 | 9.14 (4.60-18.17) | <0.001 |
| IDU |  |  |  |  |  |  |
| No IDU (ref.) |  |  |  |  |  |  |
| Daily/almost daily | 2.67 (1.50-4.75) | 0.001 | 2.58 (1.51-4.39) | <0.001 | 2.53 (1.49-4.29) | 0.001 |
| 1-2 per week | 1.93 (0.79-4.72) | 0.149 | 2.24 (0.96-5.23) | 0.063 | 2.20 (0.95-5.10) | 0.067 |
| 1-3 per month | 1.97 (0.68-5.73) | 0.210 | 2.06 (0.79-5.38) | 0.138 | 1.99 (0.77-5.16) | 0.157 |
| Polydrug use | 2.57 (1.49-4.44) | 0.001 | 2.19 (1.34-3.60) | 0.002 | 2.25(1.37-3.70) | 0.001 |
| Const. | 0.05 (0.01-0.21) | <0.001 | 0.07 (0.02-0.22) | <0.001 | 0.06 (0.02-0.21) | <0.001 |

*Previous imprisoned, number of previous imprisonments, substance use related crime, number of convictions in baseline sentence, length of baseline imprisonment and age at first imprisonment.

DAG


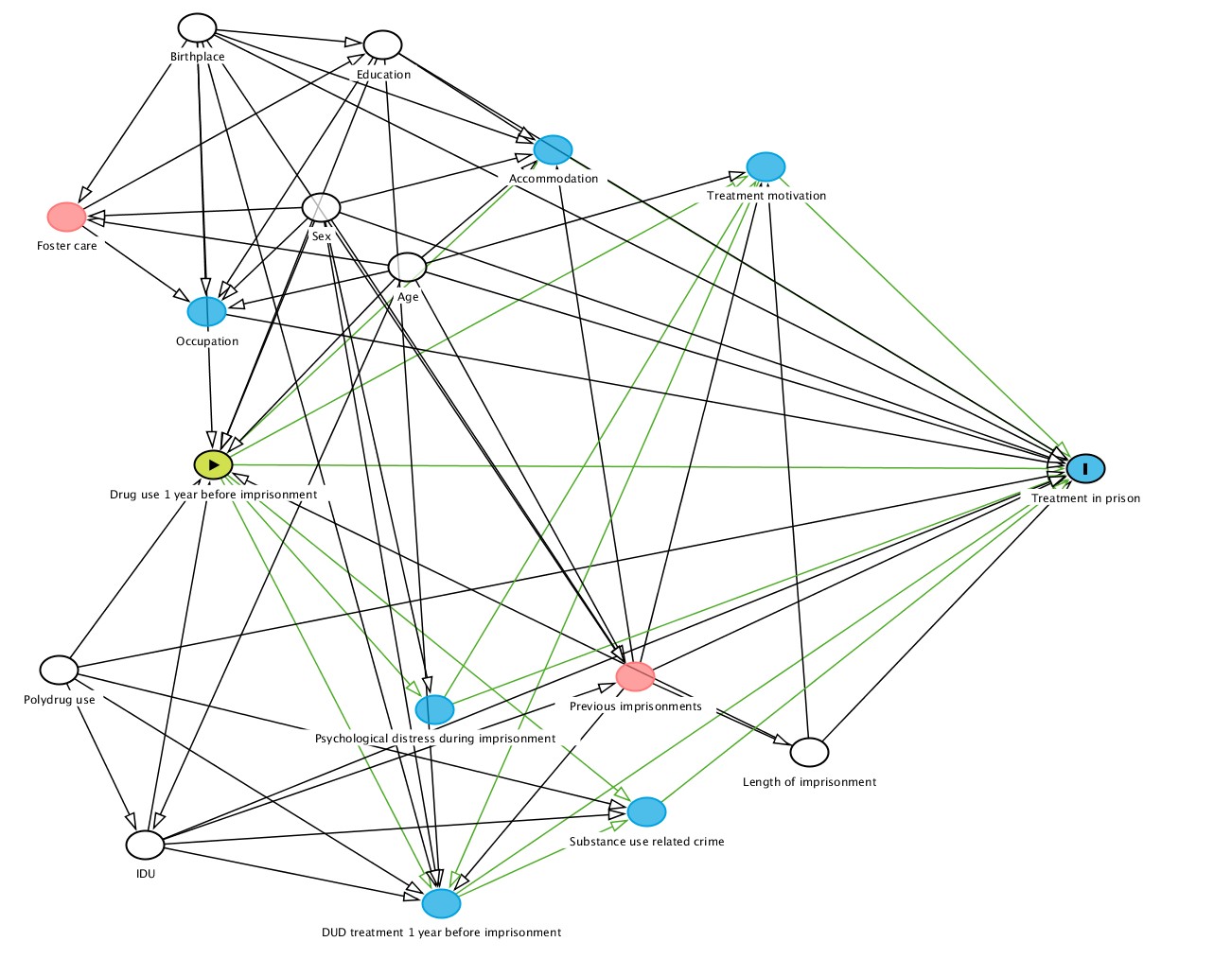


Directed Acyclic Graph constructed with Dagitty.net using the following code:

dag {

bb="-0.5,-0.5,0.5,0.5"

"DUD treatment 1 year before imprisonment" [pos="-0.142,0.442"]

"Drug use 1 year before imprisonment" [exposure,pos="-0.327,-0.015"]

"Foster care" [pos="-0.446,-0.274"]

"Length of imprisonment" [adjusted,pos="0.157,0.285"]

"Polydrug use" [adjusted,pos="-0.452,0.199"]

"Previous imprisonments" [pos="0.016,0.206"]

"Psychological distress during imprisonment" [pos="-0.147,0.240"]

"Substance use related crime" [pos="0.025,0.347"]

"Treatment in prison" [outcome,pos="0.381,-0.011"]

"Treatment motivation" [pos="0.122,-0.326"]

Accommodation [pos="-0.051,-0.344"]

Age [adjusted,pos="-0.169,-0.221"]

Birthplace [adjusted,pos="-0.340,-0.471"]

Education [adjusted,pos="-0.189,-0.453"]

IDU [adjusted,pos="-0.382,0.381"]

Occupation [pos="-0.332,-0.175"]

Sex [adjusted,pos="-0.239,-0.284"]

"DUD treatment 1 year before imprisonment" -> "Substance use related crime"

"DUD treatment 1 year before imprisonment" -> "Treatment in prison"

"Drug use 1 year before imprisonment" -> "DUD treatment 1 year before imprisonment"

"Drug use 1 year before imprisonment" -> "Psychological distress during imprisonment"

"Drug use 1 year before imprisonment" -> "Substance use related crime"

"Drug use 1 year before imprisonment" -> "Treatment in prison"

"Drug use 1 year before imprisonment" -> "Treatment motivation"

"Drug use 1 year before imprisonment" -> Accommodation

"Foster care" -> Education

"Foster care" -> Occupation

"Length of imprisonment" -> "Drug use 1 year before imprisonment"

"Length of imprisonment" -> "Treatment in prison"

"Length of imprisonment" -> "Treatment motivation"

"Polydrug use" -> "DUD treatment 1 year before imprisonment"

"Polydrug use" -> "Drug use 1 year before imprisonment"

"Polydrug use" -> "Substance use related crime"

"Polydrug use" -> "Treatment in prison"

"Polydrug use" -> IDU

"Previous imprisonments" -> "DUD treatment 1 year before imprisonment"

"Previous imprisonments" -> "Length of imprisonment"

"Previous imprisonments" -> "Treatment in prison"

"Previous imprisonments" -> "Treatment motivation"

"Previous imprisonments" -> Accommodation

"Psychological distress during imprisonment" -> "Treatment in prison"

"Psychological distress during imprisonment" -> "Treatment motivation"

"Substance use related crime" -> "Treatment in prison"

"Treatment motivation" -> "DUD treatment 1 year before imprisonment"

"Treatment motivation" -> "Treatment in prison"

Accommodation -> "Treatment in prison"

Age -> "Drug use 1 year before imprisonment"

Age -> "Foster care"

Age -> "Previous imprisonments"

Age -> "Treatment in prison"

Age -> "Treatment motivation"

Age -> Accommodation

Age -> IDU

Age -> Occupation

Birthplace -> "DUD treatment 1 year before imprisonment"

Birthplace -> "Drug use 1 year before imprisonment"

Birthplace -> "Foster care"

Birthplace -> "Previous imprisonments"

Birthplace -> "Treatment in prison"

Birthplace -> Accommodation

Birthplace -> Education

Birthplace -> Occupation

Education -> "DUD treatment 1 year before imprisonment"

Education -> "Drug use 1 year before imprisonment"

Education -> "Treatment in prison"

Education -> Accommodation

Education -> Occupation

IDU -> "DUD treatment 1 year before imprisonment"

IDU -> "Drug use 1 year before imprisonment"

IDU -> "Previous imprisonments"

IDU -> "Substance use related crime"

IDU -> "Treatment in prison"

Occupation -> "Treatment in prison"

Sex -> "DUD treatment 1 year before imprisonment"

Sex -> "Drug use 1 year before imprisonment"

Sex -> "Foster care"

Sex -> "Previous imprisonments"

Sex -> "Psychological distress during imprisonment"

Sex -> "Treatment in prison"

Sex -> Accommodation

Sex -> Occupation}

**Supplementary table 2: Logistic regression model on treatment status, including gender**

| **Outcome:**  Treatment (any treatment /no treatment) |  | |
| --- | --- | --- |
|  | aOR (95 % CI) | *p* |
| Female | 1.01 (0.44-2.33) | 0.973 |
| Nordic born | **2.85 (1.42-5.73)** | **0.003** |
| Education: More than primary school | 0.73 (0.48-1.11) | 0.140 |
| Age at baseline | 0.98 (0.96-1.00) | 0.103 |
| Length of imprisonment |  | |
| 3-6 | **3.45 (1.58-7.53)** | **0.002** |
| 6-12 | **6.34 (3.09-13.02)** | **<0.001** |
| >12 | **8.89 (4.45-17.77)** | **<0.001** |
| IDU |  | |
| Daily/almost daily | **2.58 (1.52-4.39)** | **<0.001** |
| 1-2 per week | 2.24 (0.96-5.25) | 0.064 |
| 1-3 per month | 2.06 (0.79-5.38) | 0.139 |
| Polydrug use | **2.19 (1.34-3.60)** | **0.002** |
| Cons. | 0.07 (0.02-0.22) | <0.001 |

Logistic regression model estimates based on pooled, imputed data, giving unadjusted and adjusted odds ratios (OR and aOR), 95 % confidence intervals (CI) and p-values. Sample of cohort participants with harmful drug use or likely dependence before imprisonment (n=483)

## REFERENCES

Berman, A. H., Palmstierna, T., Kallmen, H., & Bergman, H. (2007). The self-report Drug Use Disorders Identification Test: Extended (DUDIT-E): reliability, validity, and motivational index. *Journal of Substance Abuse Treatment, 32*(4), 357-369. doi:10.1016/j.jsat.2006.10.001

Derogatis, L. R., Lipman, R. S., Rickels, K., Uhlenhuth, E. H., & Covi, L. (1974). The Hopkins Symptom Checklist (HSCL): a self-report symptom inventory. *Behav Sci, 19*(1), 1-15.
